# Supplementary material for: End Sequence Analysis Toolkit (ESAT) expands the extractable information from single-cell RNA-seq data
Source: Genome Res. 2016 Oct;26(10):1397–410. doi: 10.1101/gr.207902.116 (PMC5052061; doi:10.1101/gr.207902.116)
Supplement: Supplemental Material [file supp_gr.207902.116_Supp_Materials.tar.gz › ESAT-esat_v0.1/doc/html/classumms_1_1core_1_1alignment_1_1_smatch_like-members.html]

ESAT: Member List


ESAT


- Main Page
- Related Pages
- Packages
- Classes
- Files

- Class List
- Class Index
- Class Hierarchy
- Class Members

All Classes Namespaces Files Functions Variables Enumerator Pages

- umms
- core
- alignment
- SmatchLike

umms.core.alignment.SmatchLike Member List

This is the complete list of members for umms.core.alignment.SmatchLike, including all inherited members.

|  |  |  |
| --- | --- | --- |
| getAllPossibleTargets(int numMismatches) | umms.core.alignment.SmatchLike |  |
| getForwardTargetRegions(int numMismatches) | umms.core.alignment.SmatchLike |  |
| getForwardTargets(int numMismatches) | umms.core.alignment.SmatchLike |  |
| getReverseTargets(int numMismatches) | umms.core.alignment.SmatchLike |  |
| main(String[] args) | umms.core.alignment.SmatchLike | static |
| numPossibleTargets() | umms.core.alignment.SmatchLike |  |
| SmatchLike(String kmer, List< Sequence > geneSequences, int seedNum) | umms.core.alignment.SmatchLike |  |
| SmatchLike(String kmer, String primer, int seedNum) | umms.core.alignment.SmatchLike |  |
| SmatchLike(String kmer2, Collection< Pair< String >> tailPrimers, Pair< String > subprimer1) | umms.core.alignment.SmatchLike |  |


---

Generated on Fri Aug 15 2014 15:11:07 for ESAT by  

 1.8.7
